# Successful desensitization of Pegvaliase (Palynziq®) in a patient with phenylketonuria

**DOI:** 10.1016/j.ymgmr.2020.100575

**Published:** 2020-03-06

**Authors:** Meera Patrawala, Merin Kuruvilla, Hong Li

**Affiliations:** a Division of Pulmonary, Allergy, Critical Care and Sleep Medicine, Emory University, Atlanta, GA, United States of America; b Department of Human Genetics, Emory University, School of Medicine, Atlanta, GA, United States of America; c Department of Pediatrics, Children's Healthcare of Atlanta, Atlanta, GA, United States of America

## Abstract

Pegvaliase (Palynziq®) was FDA approved in 2018 as an enzyme substitution therapy in patients with Phenylketonuria. However, various drug induced hypersensitivity adverse events (HAEs) have been reported. We present a case of Pegvaliase (Palynziq®) induced anaphylaxis and successful desensitization. A 13-step desensitization protocol was performed using three solution concentrations of Palynziq with premedication of diphenhydramine and prednisone in an outpatient setting. The patient tolerated the desensitization and was able to continue Palynziq.

## 1 Background

Phenylketonuria (PKU) is an autosomal recessive disorder caused by a deficiency of phenylalanine hydroxylase (PAH), which converts the amino acid phenylalanine into tyrosine. In patients with PKU, excessive levels of phenylalanine (PHE) can lead to neurological, cognitive, developmental and psychiatric problems. Historically, the dietary treatment alone or combined with sapropterin dihydrochloride (Kuvan®) has been used to lower the PHE levels. The poor compliance with diet or lack of responsiveness to Kuvan in some patients limited the treatment success. Studies have shown 80% of those aged 15 years and older have had PHE concentrations above the recommendation, suggesting it is difficult for adults to maintain the recommended concentration of PHE [1]. In 2018, the FDA approved Pegvaliase-pqpz (Palynziq®), the first treatment that lowers the blood PHE level by substituting for the PAH enzyme and offers the possibility to normalize the diet. However, various HAEs may occur, ranging from local injection site reactions to systemic anaphylaxis. There is currently no desensitization protocol published for this newly approved pegvaliase-pqpz in the scenario of anaphylaxis. We present a 13-step desensitization procedure, which was successfully implemented in the outpatient setting.

## 2 Case presentation

A 26-year-old Caucasian female with PKU presented after developing severe anaphylaxis to Palynziq. She had slowly titrated to daily 10 mg doses of subcutaneous Palynziq without premedication over three months. However, two weeks after initiation of 10 mg daily dose, she developed generalized pruritus, diffuse urticaria, angioedema of the face, diaphoresis, and emesis within a few minutes after receiving her injection. She self-administered epinephrine at home and was evaluated at a local emergency department. Her presentation was consistent with grade III anaphylaxis. Based on data from the Pegvaliase clinical trial program, the current recommendation for less severe systemic hypersensitivity reaction defined by grade 1 or 2 anaphylaxis, rechallenge can be considered at a lower dose or frequency in a controlled medical setting. However, in the case of severe reactions defined by grade 3 anaphylaxis as our patient experienced, the recommendation is to consider permanently discontinuing the medication [2]. Given the notable benefit of the medication, she was referred to allergy for possible desensitization. After a discussion of the risks and benefits, she was scheduled to undergo desensitization.

## 3 Method and results

A well-described 13-step desensitization protocol was performed using three solution concentrations of Palynziq. Based on her required therapeutic dose (10 mg) at that time, solutions of 0.04 mg/mL, 0.4 mg/mL, and 20 mg/mL were prepared. The patient was pre-medicated with diphenhydramine 25 mg and prednisone 40 mg the morning of her desensitization. She successfully underwent an in-office desensitization as outlined in Table 1 without any complications [3]. To date, she successfully tolerated an escalation in her dose to 20 mg daily subcutaneous without incident.

**Table 1:** Desensitization protocol

Total dose	10 mg	Solution concentration	Total dose in each solution (mg)
Solution A	5 mL	0.04 mg/mL	0.2 mg
Solution B	5 mL	0.4 mg/mL	2 mg
Solution C	1 mL	20 mg/mL	20 mg


Step	Solution	Administered volume (mL)	Time (min)	Administered dose (mg)	Fold increase per step	Approximate cumulative dose (mg)
1	A	0.5	15	0.02	NA	0.02
2	A	1	15	0.04	X2	0.06
3	A	1.5	15	0.06	X1.5	0.12
4	A	2	15	0.08	x2	0.2
5	B	0.25	15	0.1	X1.2	0.3
6	B	0.5	15	0.2	X2	0.5
7	B	1	15	0.4	X2	0.9
8	B	1.5	15	0.6	X1.5	1.5
9	B	1.75	15	0.7	X1.5	2.2
10	C	0.05	15	1	X1.5	3.2
11	C	0.1	15	2	X2	4.2
12	C	0.1	15	2	N/A	6.2
13	C	0.2	120	4	X2	10.2

## 4 Discussion

While a spectrum of hypersensitivity reactions may occur with pegvaliase-pqpz, acute systemic hypersensitivity reactions develop in 0.02%–0.08% of patients [4]. These are thought to be caused by Polyethylene glycols (PEGs), polyether compounds used in medical products to prolong blood circulation half-lives, improve drug solubility and stability, and reduce immunogenicity. Although thought to be biologically inert, PEGs have been suspected as the culprit in many hypersensitivity reactions. A recent review of published cases by Garvey et al. reported 37 cases of hypersensitivity to PEG in the past four decades [5]. The mechanism underlying PEG hypersensitivity is not well understood, and anti-PEG specific IgE measurement is still experimental. With this medication, the mechanism of action described has been immune complex-mediated type III hypersensitivity related to binding of anti-drug antibodies to pegvaliase leading to circulating immune complex formation and complement activation [6].

To our knowledge, this is the first reported desensitization of severe anaphylaxis to Pegvaliase. In the appropriate clinical context, such as patients with more severe hypersensitivity reactions, desensitization may be another approach to management of these patients in order to continue the effective treatment.

## Author statement

All authors have contributed to the drafting and revision of the manuscript. There are no conflicts of interest.
